# Risk prediction model for epithelial ovarian cancer using molecular markers and clinical characteristics

**DOI:** 10.1186/s13048-015-0195-6

**Published:** 2015-10-21

**Authors:** Meiying Zhang, Guanglei Zhuang, Xiangjun Sun, Yanying Shen, Aimin Zhao, Wen Di

**Affiliations:** Department of Obstetrics and Gynecology, Ren Ji Hospital, School of Medicine, Shanghai Jiao Tong University, 160 Pujian Road, Shanghai, 200127 China; Shanghai Key Laboratory of Gynecologic Oncology, Ren Ji Hospital, School of Medicine, Shanghai Jiao Tong University, 160 Pujian Road, Shanghai, 200127 China; State Key Laboratory of Oncogenes and Related Genes, Renji-Med X Clinical Stem Cell Research Center, Ren Ji Hospital, School of Medicine, Shanghai Jiao Tong University, 160 Pujian Road, Shanghai, 200127 China; Department of Pathology, Ren Ji Hospital, School of Medicine, Shanghai Jiao Tong University, 160 Pujian Road, Shanghai, 200127 China

**Keywords:** Epithelial ovarian cancer, Risk prediction model, Molecular classification, Overall survival

## Abstract

**Background:**

A high-quality risk prediction model is urgently needed for the clinical management of ovarian cancer. However most existing models are solely based on clinical parameters, and molecular classifications in recent reports are still being debated. This study aimed to establish a risk prediction model by using both clinicopathological and molecular factors (the synthetic model) for epithelial ovarian cancer.

**Methods:**

A retrospective cohort study was conducted in epithelial ovarian cancer patients (*n* = 161) treated with primary debulking surgery and adjuvant chemotherapy. The expression level of 15 selected molecular markers were measured using immunohistochemistry. A risk model was developed using COX regression analysis with overall survival as the primary outcome. A simplified scoring system for each prognostic factor was based on its coefficient. Independent validation (*n* = 40) was conducted to evaluate the performance of the model.

**Results:**

A total of 10 out of 15 molecular markers were significantly associated with clinical characteristics and overall survival. The synthetic model performed better than the clinicopathological risk model or the molecular risk model alone, as assessed by analysis of the receiver-operating characteristics curve area and the Youden index. The synthetic model included parity (>3), peritoneal metastasis, stage, tumor type, residual disease, and expression of human epidermal growth factor receptor 2 (HER2), epidermal growth factor receptor (EGFR), breast cancer 1 (BRCA1), murine sarcoma viral oncogene homolog B (BRAF) and Kirsten rat sarcoma viral oncogene homolog (KRAS).

**Conclusions:**

Our synthetic risk model may more accurately predict survival of epithelial ovarian cancer patients than current models.

**Electronic supplementary material:**

The online version of this article (doi:10.1186/s13048-015-0195-6) contains supplementary material, which is available to authorized users.

## Background

Risk prediction in patients with intractable disease is one of the major challenges for both clinic treatment and basic research, which tailors risk assessment of outcome based on the individual’s clinical, epidemiological and molecular factors [1]. By means of the risk prediction model, disease can be more efficiently monitored and precisely treated. Ovarian cancer is the most lethal gynaecologic malignancy. Despite improvements in diagnostic methods, surgical technologies and chemotherapeutic agents over the past few years, the prognosis is still poor [2–5]. Therefore, the development of a high-quality risk prediction model for ovarian cancer to guide personalized therapy is a primary research focus in the field.

In 1989, Van Houwelingen first reported a prognostic index (PI) for ovarian cancer [6]. Since then, a series of studies reported that the prognostic model, which is based on clinical characteristics including advanced age, higher stage and grade of tumor, presence of ascites, poorer performance status and residual disease (>1 cm), was able to stratify patients with poor survival [7–9]. However, ovarian cancer patients with similar clinical characteristics also exhibit difference in prognosis, which may be due to high molecular heterogeneity of tumor and/or different molecular genetics [10, 11]. It has been reported that expression or status of certain molecular markers, such as tumor protein 53 (TP53), epidermal growth factor receptor (EGFR), myelin and lymphocyte protein (MAL), and breast cancer 1/2 (BRCA1/BRCA2) are independent predictors of patient survival in serous ovarian cancer [12–14]. However, the accuracy of these gene signatures remains controversial, because previous studies are merely on serous ovarian cancer without validation datasets.

Compared with other molecular detection techniques, immunohistochemistry has its own advantages: lower cost, presenting morphology for cells and tissues and less time consuming. The aim of the current study was to elucidate epithelial ovarian cancer (EOC) at the molecular level and to establish a predictive model for EOC using immunohistochemistry. To consider cancer heterogeneity and to improve prognostic accuracy, we further integrated molecular and clinicopathological factors into the risk model.

## Methods

### Patients

Our research has been approved by Ethics Committee of Ren Ji Hospital, Shanghai Jiao Tong University, School of Medicine, and informed consents were obtained from all epithelial ovarian cancer patients or their direct relatives. The tumor tissue specimens were collected from patients who were operated and confirmed with histopathology post-operation between June 2003 and December 2009 in the Department of Obstetrics and Gynaecology, Ren Ji Hospital, Shanghai, China. The subjects were divided into two groups: an experimental group (*n* = 161) and a validation group (*n* = 40). Each patient had undergone cytoreductive surgery (without neoadjuvant chemotherapy, NACT) and a standardized post-surgical course of chemotherapy based on platinum. The following factors were recorded for the experimental group (*n* = 161): the clinicopathological characteristics (Table 1), the effect of chemotherapy based on the platinum (Table 1) and follow-up outcome. Platinum-resistant ovarian cancer (PROC) was defined as recurrence within 6 months of the completion of platinum-based chemotherapy and disease progression within 6 months during or after chemotherapy [15].

**Table 1:** Patient characteristics and potential prognostic factors

Characteristics	*N*	(%)	Median survival (months)	*P* ^b^
Age				0.095
<40	7	4.3	83.0	
40–49	31	19.3	33.1	
50–59	62	38.5	36.0	
60–69	33	20.5	46.0	
≥70	26	16.1	60.3	
Parity				0.019*
0–1	77	47.8	62.2	
2–3	66	41.0	52.0	
>3	15	9.3	45.0	
Menopause				0.716
Yes	106	65.8	45.0	
No	53	32.9	49.4	
Ascites				0.735
Yes	62	38.5	37.0	
No	97	60.2	59.5	
Peritoneal metastasis				0.004^*^
Yes	89	55.3	31.6	
No	71	44.1	60.0	
Lymphatic metastasis				0.905
Yes	69	42.9	26.3	
No	91	56.5	58.6	
FIGO stage				0.025^*^
I	55	34.2	62.1	
II	18	11.2	42.0	
III	80	49.7	35.0	
IV	7	4.3	18.3	
Histotype				0.954
Serous	114	70.8	38.7	
Mucinous	15	9.3	63.0	
Endometrioid	13	8.1	59.0	
Clear cell	9	5.6	60.4	
Undifferentiated	10	6.2	29.0	
Grade				0.415
G1	33	20.5	61.0	
G2	57	35.4	55.0	
G3	69	42.9	30.1	
Tumor type^a^				0.003^*^
I	57	35.4	61.0	
II	101	62.7	36.0	
Residual disease				<0.0001^*^
≤0.5 cm	119	73.9	58.0	
>0.5 cm	40	24.8	19.4	
Platinum resistance				<0.0001^*^
Yes	36	22.4	22.0	
No	123	76.4	58.0	

^*^statistical significance

^a^Based on morphological and molecular genetic analysis, EOC are divided into two categories: type I tends to be low-grade neoplasms; while type II is high-grade neoplasms [35]

^b^Log-rank test

### Immunohistochemistry

Using immunohistochemistry, 15 molecular markers were selected for analysis in specimens after evaluating a range of published prognostic molecular markers [11, 12, 16–22] (Additional file 1: Table S1) and considering the tumor characteristics [23]. The immunohistochemistry was performed as following: briefly, paraffin-embedded tumor specimens were antigen retrieved in a microwave at >90 °C for 15 min after dewaxing and rehydration, then were blocked with 5 % bovine serum albumin (BSA) for 1 h to reduce non-specific binding. The specimens were incubated sequentially with a rabbit anti-BRCA1 polyclonal antibody (SANTA CRUZ, Dallas, Texas, USA), a mouse anti-P53 monoclonal antibody (SANTA CRUZ, Dallas, Texas, USA), a rabbit anti-human epidermal growth factor receptor 2 (HER2) polyclonal antibody (SANTA CRUZ, Dallas, Texas, USA), a mouse anti-murine sarcoma viral oncogene homolog B (BRAF) monoclonal antibody (SANTA CRUZ, Dallas, Texas, USA), a mouse anti-Kirsten rat sarcoma viral oncogene homolog (KRAS) monoclonal antibody (MILLIPORE, Billerica, Massachusetts, USA), a rabbit anti-Ki67 polyclonal antibody (SANTA CRUZ, Dallas, Texas, USA), a rabbit anti-vascular endothelial growth factor (VEGF) polyclonal antibody (SANTA CRUZ, Dallas, Texas, USA), a rabbit anti-Notch homolog 3 (NOTCH3) polyclonal antibody (SANTA CRUZ, Dallas, Texas, USA), a rabbit anti-cyclin E1 (CCNE1) polyclonal antibody (SANTA CRUZ, Dallas, Texas, USA), a rabbit anti-erythroid transcription factor (GATA2) polyclonal antibody (SANTA CRUZ, Dallas, Texas, USA), a rabbit anti-forkhead box protein M1 (FOXM1) polyclonal antibody (SANTA CRUZ, Dallas, Texas, USA), a rabbit anti-BRCA2 polyclonal antibody (SANTA CRUZ, Dallas, Texas, USA), a rabbit anti-EGFR polyclonal antibody (SANTA CRUZ, Dallas, Texas, USA), a rabbit anti-phosphatase and tensin homolog (PTEN) polyclonal antibody (Zhongshan, Beijing, China), a mouse anti-multidrug resistance (MDR1) monoclonal antibody (SANTA CRUZ, Dallas, Texas, USA) for 2 h at 1:100 dilution and a horseradish peroxidase (HRP)-conjugated goat anti-mouse or anti-rabbit IgG antibody (Zhongshan, Beijing, China; 1:100) for 1 h. 3,3′-diaminobenzidine tetrahydrochloride (DAB; Zhongshan, Beijing, China) and hematoxylin were used to colour the slides. The semiquantitative evaluation of the staining for 15 molecular markers was performed by two pathologists in blind fashion, as described in the Additional file 1 [12, 24–32].

### Statistical analysis

The outcome was the survival time from diagnosis to the date of death. The surviving patients’ cut-off was the date of the last follow-up if the duration of follow-up was more than 5 years.

Multiple imputations have been advocated as an appropriate method to manage missing data [33]. The sequential regression multiple imputation (SRMI) method, serving as an imputation model, was applied to make up the missing data. We then used the multiple-imputed data sets to analyse the variables [34].

SPSS 19.0 software (IBM, Armonk, New York, USA) was used for the analyses. The differences in survival according to clinicopathological and 15 molecular factors were assessed by using a log-rank test and a univariate analysis. We established the risk model of survival via multivariate COX regression and estimated hazard rate (HR). Each risk factor’s score was calculated from beta-coefficient in the multivariate analysis. The risk model for every patient was the sum of the scores of each factor. The receiver-operating characteristics (ROC) curve area and the Youden index were then utilized to calculate the cut-off points of the risk model. Based on the cut-off points, the risk model of survival was divided into two classes: low risk and high risk. We then adopted an independent validation in the validation group and used the Kaplan-Meier method to analyse the difference between the two risk classes. For all analyses, *P* < 0.05 was considered significant.

## Results

### Description of the study cohort

The clinicopathological characteristics of the experimental group (*n* = 161) were presented in Table 1. The median survival was 48 months (range: 8–123 months); the international federation of gynaecology and obstetrics (FIGO) stage was predominantly III (49.7 %) and I (34.2 %) at initial diagnosis; and the histopathology mainly exhibited serous epithelial ovarian cancer (70.8 %); the proportions of other pathological subtypes (mucinous, endometrioid, clear cell and undifferentiated cancer) were similar; 73.9 % patients underwent the ideal tumor reductive surgery.

### Predictors of survival

#### Clinicopathological characteristics

**Fig. 1:**
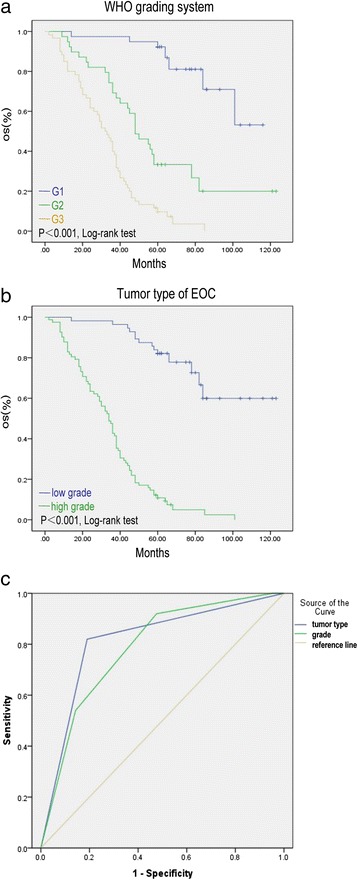
Kaplan-Meier survival curve and ROC curve of WHO grading system and tumor type. **a** Kaplan-Meier survival curve of WHO grading system. **b** Kaplan-Meier survival curve of tumor type. **c** ROC curve of WHO grading systems and tumor type. The area was 0.815 (blue line: tumor type) vs. 0.787 (green line: WHO grade)

Based on the univariate analyses of clinicopathological characteristics, six variables, including parity, peritoneal metastasis, FIGO stage, tumor type [35], residual disease and platinum resistance were statistically significant risk factors for overall survival (*P* ≤ 0.05). Overall survival was more significantly associated with the tumor type than with the WHO grade (the area under the ROC curve was 0.815 vs. 0.787) (Fig. 1).

#### Molecular markers

The immunohistochemistry results of the molecular markers were presented in Additional file 1: Figure S1. Using the Kaplan-Meier survival analysis, 10 molecular markers were identified to be significant correlated with overall survival (Additional file 1: Figure S2).

The relationships between the molecular markers and the clinicopathological features were illustrated in Additional file 1: Table S2. The patients with strong staining or complete absence of P53 expression, which presumably lost P53 function (Strong staining or complete absence of P53 expression is defined as *TP53* mutation) tended to display increased peritoneal metastasis (*P* = 0.027) and later FIGO stage (*P* = 0.024). The tumor type was closely associated with the expression of P53 (*P* = 0.002). The strong staining or complete absence of P53 which is equivalent to loss of P53 function was most frequently observed in serous carcinomas (70.8 %). The significant association between high HER2 expression and the tumor type (*P* = 0.025) as well as with residual disease (*P* = 0.002) was observed. Strong staining of KRAS (the *KRAS* mutation commonly had strong staining) in the tumor cell nuclei was significantly associated with FIGO stage (*P* = 0.011), tumor type (*P* = 0.025) and WHO grade (*P* = 0.027). The absence or weak staining of BRCA1 which was considered as *BRCA1* mutation, was also significantly associated with tumor type (*P* = 0.048). The absence or weak staining of BRAF (Negative and weak staining in tumor cell cytoplasm was considered to be mutation *BRAF*) (*P* = 0.045) and high EGFR expression (*P* = 0.041) were associated with the ovarian cancer histotype. Serous (69.5 %) and mucinous (11.3 %) ovarian cancer had high proportions of low BRAF expression, while serous (63.6 %) and endometrioid (13.6 %) ovarian cancer exhibited high EGFR expression. Two of the most striking findings were that (a) highly significant associations were discovered between the overexpression of HER2 (26/36 resistant patients, *P* = 0.013), KRAS (32/36 resistant patients, *P* = 0.004), low expression of PTEN (36/36 resistant patients, *P* = 0.043) and platinum resistance, and (b) platinum resistance (*P* = 0.043), residual disease (*P* < 0.001), the expression of VEGF (*P* = 0.031) and HER2(*P* = 0.008), the expression of BRCA1(*P* = 0.05) and KRAS (*P* = 0.021) were significantly associated with overall survival in patients with the same stages and treated with uniform therapies. There were no associations between clinicopathological characteristics and the expression of VEGF, NOTCH3, and BRCA2.

### The risk models

To evaluate the association between ovarian cancer patient outcomes and the clinicopathological characteristics as well as molecular markers, we established three risk models: the clinicopathological model, the molecular model and the synthetic model comprising both clinicopathological characteristics and molecular markers. First, we performed a multivariate COX regression analysis to independently assess the relationship between clinical characteristics or molecular markers and patient survival. The results showed that parity, peritoneal metastasis, FIGO stage, tumor type and residual disease were independent prognostic factors among the clinical characteristics (Table 2). Among the molecular markers, HER2, KRAS, BRCA1, BRAF, and EGFR were independent and statistically significant prognostic factors for ovarian cancer patient outcomes (Table 2). The risk score of each predictor was obtained from the coefficient of the COX regression model. Consequently, the total score for the clinicopathological model was 7 points, while the total score for the molecular model was 5 points. The score for the synthetic model was generated using the sum of the scores of the above independent clinicopathological and molecular factors (12 points). The three scoring systems were shown in Table 3.

**Table 2:** The Cox regression analysis of prognostic factors in epithelial ovarian cancer

Factors	β	Hazard ratio	95 % confidence interval	*P*
Clinicopathological factors				
Parity				0.009
0–1			Reference	
2–3	0.103	0.668	0.410–1.089	
>3	1.069	2.911	1.203–7.044	
Peritoneal metastasis				0.047
No			Reference	
Yes	1.086	2.963	1.013–8.664	
FIGO stage				0.021
I			Reference	
II	0.722	2.058	0.603–7.025	
III	1.150	3.158	0.730–13.658	
IV	1.470	4.351	1.611–11.746	
Tumor type				<0.001
I			Reference	
II	1.613	5.017	2.501–10.067	
Residual disease				<0.001
≤0.5 cm			Reference	
>0.5 cm	1.553	4.725	2.418–9.233	
Molecular factors				
HER2				<0.001
Low expression			Reference	
High expression	1.242	3.463	1.839–6.523	
KRAS				<0.001
Low expression			Reference	
High expression	1.332	3.787	1.959–7.319	
BRCA1				0.003
High expression			Reference	
Low expression	0.957	2.604	1.398–4.849	
BRAF				0.012
High expression			Reference	
Low expression	1.043	2.838	1.261–6.383	
EGFR				0.036
Low expression			Reference	
High expression	0.622	1.862	1.042–3.327

**Table 3:** Scoring system for the three risk models in epithelial ovarian cancer

Scoring	Impact factors										Clinicopathological model	Molecular model	Model comprising clinicopathological and molecular factors
	Parity	Peritoneal metastasis	FIGO stage	Type	Residual disease	HER2 expression	KRAS expression	BRCA1 expression	BRAF expression	EGFR expression			
0	0–3	Absent	I	I	≤0.5 cm	Low	Low	High	High	Low			
1	>3	Present	II–IV			High	High	Low	Low	High			
2				II	>0.5 cm								
Total score											7	5	12

**Fig. 2:**
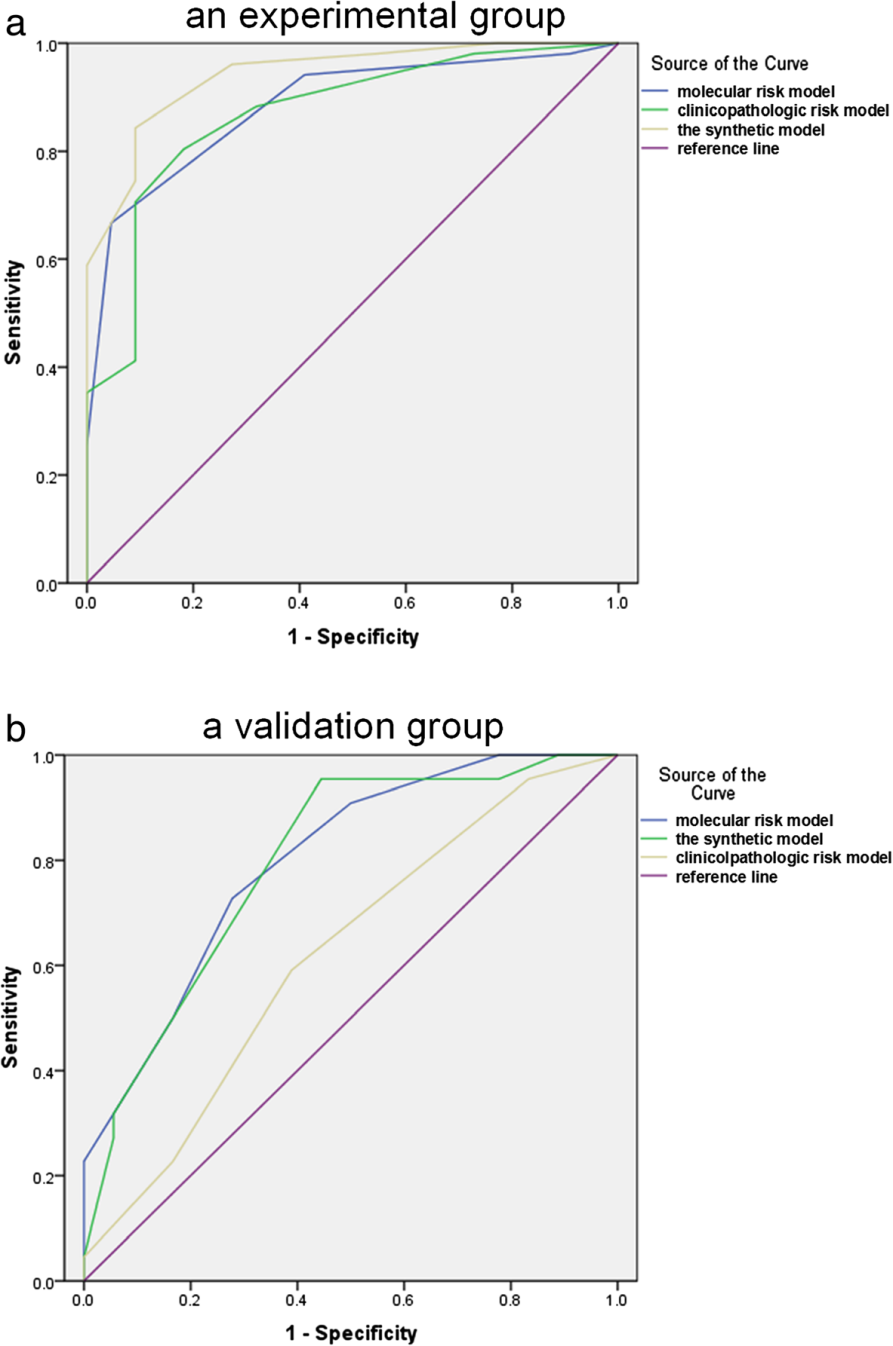
ROC curves of the three risk models in the experimental group and the validation group. **a** In the experimental group, the areas under the curve were as following: the molecular risk model (blue line: 0.884), the clinicopathological risk model (green line: 0.869), and the synthetic model (yellow line: 0.942). **b** The ROC areas in the validation group were shown: the synthetic model (green line: 0.798), the clinicopathological model (yellow line: 0.620) and the molecular model (blue line: 0.794)

**Fig. 3:**
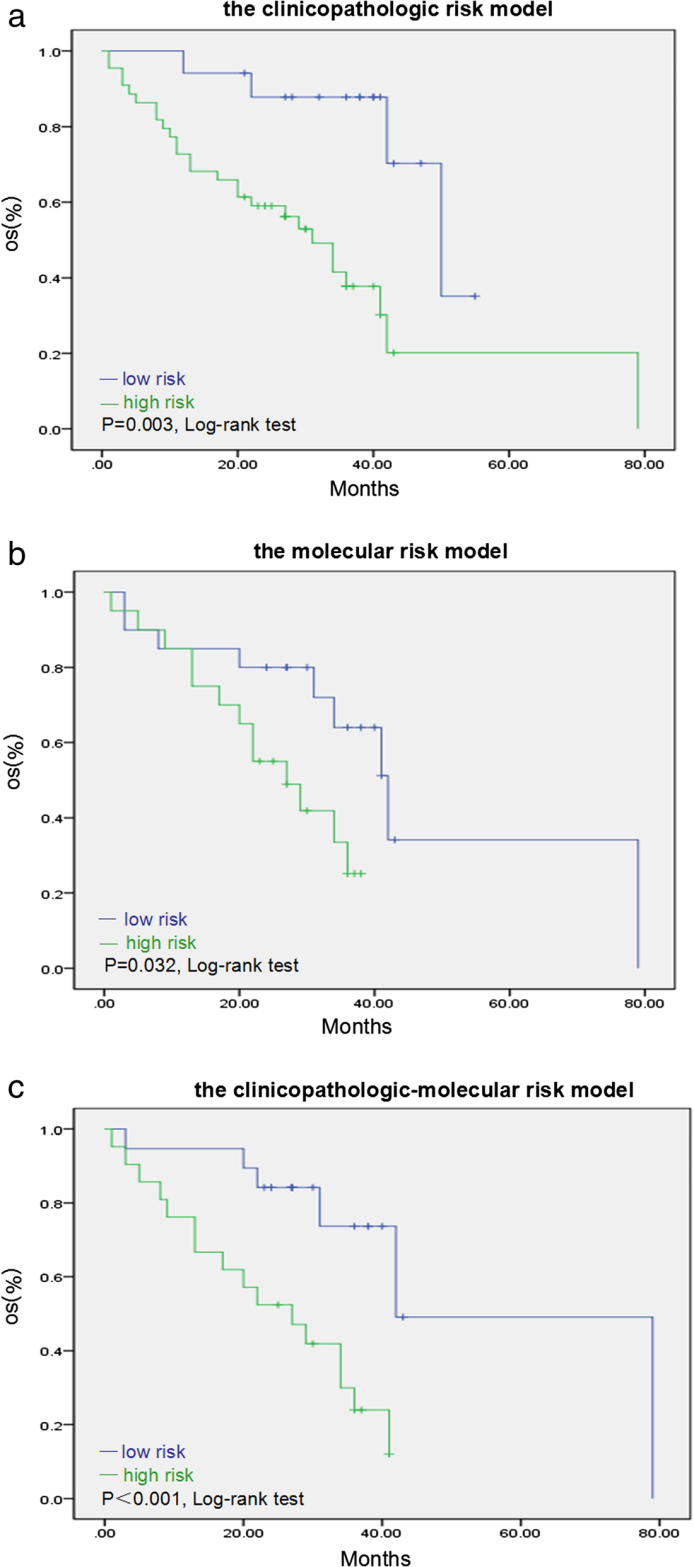
The validation of the three risk models using the Kaplan-Meier survival analysis. **a** The clinicopathological risk model. **b** The molecular risk model. **c** The clinicopathological-molecular risk model. The blue line denotes the low-risk class and the green line denotes the high-risk class

In the synthetic model, the ROC area was 0.942 with a cut-off at 6.5 points compared with the clinicopathological model (ROC area: 0.869, cut-off point: 2.5) and molecular model (ROC area: 0.884, cut-off point: 2.5) (Fig. 2a). Then we validated the three risk models for discrimination in an independent validation dataset. Likewise, the ROC area of the synthetic model (ROC area: 0.798, cut-off point: 6.5) was largest than the clinicopathological model (ROC area: 0.620, cut-off point: 2.5) and the molecular model (ROC area: 0.794, cut-off point: 2.5) (Fig. 2b). And these patients in validation group were divided into low- and high-risk classes according to the cut-off points in each model (Table 4). The differences between the two classes were significant for all three risk models (Table 4). The Kaplan-Meier survival analysis assessing the low- and high-risk classes for the three risk models was shown in Fig. 3. P value of the synthetic model was the most significant (*P* < 0.001). Moreover, the results showed that three patients with unfavorable outcomes classified as low risk by clinicopathological model were upgraded to high risk by molecular and synthetic model, and 14 patients whose survivals were close to median as high risk by clinicopathological model were downgraded to low risk by synthetic model.

**Table 4:** Performance of the three risk models in the validation group

Model	Score	No (%)	Death (%)	Median	*P* ^c^
Clinicopathological model	0–2^a^	12(30.0)	3(25.0)	38.0	
	3–7^b^	28(70.0)	19(67.9)	24.5	0.003
	Total	40	22(55.0)	27.0	
Molecular model	0–2^a^	20(50.0)	9(45.0)	30.5	
	3–5^b^	20(50.0)	13(65.0)	24.0	0.032
	Total	40	22(55.0)	27.0	
Model comprising molecular and clinicopathological factors	0–6^a^	19(47.5)	6(31.6)	30.6	
	7–12^b^	21(52.5)	16(76.2)	25.0	<0.001
	Total	40	22(55.0)	27.0	

^a^Low-risk

^b^High-risk

^c^The results were calculated using the Kaplan –Meier method

## Discussion

These molecular markers may be beneficial for predicting the outcome and the response to treatment for EOC, because these biomarkers can evaluate multiple genetic alterations, compared with the classical clinicopathological prognostic factors [36, 37]. To our best knowledge, although several molecular prognostic models for serous ovarian cancer have been established, there is not a clinical unified and widely applicable model. With respect to EOC, Carsten proposed the prognostic index system, which contained clinical pathological characteristic and 300 genes [38]. This prognostic system was considered to be a thorough genetic screening of EOC. In contrast, our synthetic model is a preliminary exploration to use only a handful of molecular biomarkers in conjunction with clinicopathological factors to generate a prognostic model for EOC. Notably, immunohistochemistry of these molecular markers has been now widely and routinely used for clinical diagnosis, and the uniform criteria for each molecule has been defined in immunohistopathology. In order to improve the detection efficiency and refrain from wasting EOC cancer specimen, we can prepare tissue microarray (TMA) with which all molecular markers can be tested on one slide. Even though immunohistochemistry is a semi-quantitative approach, we have validated its reliability by quantitative methods, such as flow cytometry in previous experiments [39]. Thus, the outcome of EOC patients after surgery can be conveniently and rapidly predicted using our model, and the individualized treatment regimen can be subsequently tested.

Importantly, our three risk models were validated in an independent cohort and could be applied to stratify patients into two risk classes. Nevertheless, the ROC curve area of the synthetic model was the largest. Using the cut-off point scoring system, the two risk classes in the synthetic model were more statistically significant than those in clinicopathological model or the molecular model. Moreover, the molecular model improved the accuracy of risk class classification which was previously classified in clinicopathological model. Our current data suggested that the biomarkers could be integrated in the risk model, which might lead to an improvement of clinicopathological stratification. Indeed, our synthetic risk model showed the best performance in estimating EOC patient survival.

Our risk models suggested that tumor type and residual disease were the strongest survival predictors, indicating the importance of the initial cytoreductive surgery to EOC outcome. The tumor type of EOC was defined by Shih based on morphological and molecular analysis [35]. Type I includes low-grade serous carcinoma, mucinous carcinoma, low-grade endometrioid carcinoma and clear cell carcinoma, which tend to be low-grade neoplasms with the mutations of *BRAF, KRAS* and *PTEN*; whereas type II is composed of high-grade serous carcinoma, high-grade endometrioid carcinoma and undifferentiated carcinoma which are high-grade neoplasms with high human leukocyte antigen-G (HLA-G) expression and *TP53* mutation as markers. The low-grade and high-grade serous carcinoma is respectively the prototypic subtype of the two types. The University of Texas M.D. Anderson Cancer Center illuminated that low- and high-grading system of the serous ovarian cancer was associated with survival and was clinically feasible [40]. This is in line with our research that tumor type is closely associated with the overall survival, and we further revealed that the tumor type classification showed a better prognostic value than the WHO grading system. With respect to residual disease, we found that a residual tumor size ≤0.5 cm was optimal for cytoreduction, which was a predictor in the COX regression model (HR: 4.725, *P* < 0.001), and the molecular marker HER2 was significantly associated with residual disease (*P* = 0.002). We also observed that other clinicopathological factors, including peritoneal metastasis and FIGO stage, were associated with EOC survival. These findings were consistent with Rutten’s model, which is the most recognized prognostic clinicopathological model for EOC patients in recent years [41]. In addition, increased parity (especially a parity >3) was associated with high EOC risk. The parity result differed from Yang’s study in which no clear association was determined between parity and ovarian cancer survival [42]. In contrast, Poule found that a long-time oral-contraception was strongly correlated with less aggressive epithelial ovarian cancer due to fewer lifetime ovulatory cycles [43]. Thus, we consider that a larger sample size will be necessary to determine whether the ovulatory and multiparous increase the risk of EOC patient outcome and try to uncover the underlying mechanism.

Gene expression signatures may predict ovarian cancer outcome only in certain subtypes of ovarian cancer, including late-stage, platinum-treated and serous ovarian cancer [14, 44, 45]. Our study sought to find molecular markers to predict overall survival for all EOC types. HER2, KRAS, BRCA1, BRAF and EGFR were validated in the risk model. EGFR and HER2 are the members of the HER family, whose EGF signaling pathway has been shown to play an important role in tumor initiation, progression and metastasis. And *KRAS* gene can regulate the signal transduction between HER receptors and the nucleus. *KRAS* mutation activates KRAS protein, which continuously stimulates the EGFR activation [46]. *BRAF* is the downstream effector of *KRAS,* which was reported to be common mutation (28–35 %) in serous borderline (SB)/low grade serous ovarian cancer (LGS-OvCa) [21]. Rachel demonstrated that LGS-OvCa patients with *BRAF* mutant were inclined to peritoneal metastasis and recurrence in the case of presence of micropapillary feature [47]. The EGFR/HER2/KRAS/BRAF signaling pathway has been reported in pancreatic cancer and colorectal cancer other than in ovarian cancer [48, 49 ]. Once mutations occur in *BRAF* and/or *KRAS*, patients are refractory to anti-EGFR therapy with poor prognosis. Skirnisdottir ever integrated EGFR into his prognostic model for early-stage EOC [12]. Our model supported notion that the four molecules in the EGFR/HER2/KRAS/BRAF signaling pathway also produce the synergic effect, exerting their own effect on ovarian cancer progression. A new developed model of prediction of EOC should take the expression of HER2 family, KRAS and BRAF into account. TCGA analysis showed that the *BRCA1* and *BRCA2* mutations in 22 % of the high grade serous ovarian cancer (HGS-OvCa) samples triggered a wide range of aberrations in DNA damage repair pathways, such as poly (ADP-ribose) polymerase inhibitors (PARPi) [11]. Besides the breast and ovarian cancer patients, some solid tumors such as prostate, lung, endometrial, pancreatic and colon cancer are also associated with *BRCA1/2* mutations [50]. And Patch observed that the germline of mutation of *BRCA1 or BRCA2* was associated with the acquired chemoresistance in HGS-OvCa [51]. In this study, our results were in line with and extend the importance of *BRCA1* mutation in EOC, which was one of the critical factors of molecular markers in our synthetic prognostic model. Therefore, *BRCA* screening is recommended to familial-risk women to prevent and diagnose EOC early. Beyond this, high HER2 expression (*P* = 0.013), high KRAS expression (*P* = 0.004) and low PTEN expression (*P* = 0.043) were associated with higher platinum chemoresistance in EOC differential from HGS-OvCa. Ovarian clear cell carcinoma (OCCC) is characterized by resistance to conventional platinum chemotherapy compared with other EOC histotypes [52]. The aberrant genes studied in OCCC included *PTEN* and *HER2* [53]. Increased HER2 may also bind to steroid receptor coactivator 3 (SRC3), which contributes to high level of malignant cell proliferation and poor survival due to platinum resistance [54]. In addition to the above observations, Patch showed that *PTEN* mutation contributed to acquired chemotherapy resistance in HGS-OvCa as well [51]. Moreover, the study by Ratner indicated that EOC patients with mutated *KRAS* were more likely to be resistant to platinum (OR = 3.18, *P* = 0.011) [55]. So far, there are several pathways by which oncogenic *KRAS* may induce chemoresistance: first, it activates the RAF/MEK/ERK pathway; secondly, *KRAS* mutation induces COX-2 expression which heightens cancer cell binding to extracellular matrix and secrets more PGE_2_ to facilitate cell migration and dissemination; thirdly, *KRAS* mutation may activate the transcription of cellular protective stress response gene nuclear factor erythroid-derived 2 (*NRF2*) to protect against oxidative damage and promote drug resistance [56–58]. LGS-OvCa harboring *KRAS* mutation is a chemoresistant disease that accounts for 10 % of serous ovarian cancer. And recurrent and chemoresistant LGS-OvCa patients were observed to be dramatic and durable responses to MEK inhibitor therapy [58]. Therefore, the KRAS/RAF/MEK/ERK pathway is now considered to be the key mechanism in chemoresistant LGS-OvCa. Interestingly, in our study of EOC with all histotypes, KRAS was still the cause of platinum chemoresistance. We envision that our findings may provide novel pathway for KRAS to induce chemoresistance that may ultimately lead to more targeted therapies.

There are some limitations in our analysis. Firstly, immunohistochemistry is the method to detect protein expression, which might not accurately assess the mutation of molecule. While the expression and function of TP53, KRAS, BRCA, BRAF are dependent on their genes status, they may better be detected by DNA sequencing to precisely elucidate these molecules’ effect on EOC prognosis. Secondly, in order to improve the model’s sensitivity and specificity, clinical multi-centre investigations are necessary to be conducted in a validation-emendation-validation manner. We would attempt to solve these problems in future studies. Once resolved, the synthetic risk model may be widely applied for clinical diagnosis of EOC.

## Conclusions

Our risk model integrating clinicopathological and molecular factors was validated to predict the overall survival of EOC patients. The information obtained from the synthetic model may assist in the development of individualized and targeted therapies.

## Additional file

Additional file 1:
**Table S1.** Prognostic molecular markers in published studies. **Table S2.** The relationship between molecular markers and clinicopathologic characteristics. **Figure S1.** Single immunostaining of 15 molecular markers in epithelial ovarian cancer. **Figure S2.** Kaplan-Meier overall survival analyses of 15 molecular markers inepithelial ovarian cancer. (ZIP 108 kb)

## Abbreviations

EOC: Epithelial ovarian cancer
NACT: Neoadjuvant chemotherapy
PROC: Platinum-resistant ovarian cancer
SRMI: Sequential regression multiple imputation
ROC: Receiver-operating characteristics
HGS-OvCa: High grade serous ovarian cancer
LGS-OvCa: Low grade serous ovarian cancer
OCCC: Ovarian clear cell carcinoma
