# Supplementary material for: Risk prediction model for epithelial ovarian cancer using molecular markers and clinical characteristics
Source: J Ovarian Res. 2015 Oct 21;8:67. doi: 10.1186/s13048-015-0195-6 (PMC4618052; doi:10.1186/s13048-015-0195-6)
Supplement: Additional file 1: — Table S1. Prognostic molecular markers in published studies. Table S2. The relationship between molecular markers and clinicopathologic characteristics. Figure S1. Single immunostaining of 15 molecular markers in epithelial ovarian cancer. Figure S2. Kaplan-Meier overall survival analyses of 15 molecular markers inepithelial ovarian cancer. (ZIP 108 kb) [file 13048_2015_195_MOESM1_ESM.zip › UPDATED ADDITIONAL FILE/Additional file.pdf]

Additional Files:

Table S1. Prognostic molecular markers in published studies

Database	Reference	Molecular markers
TCGA 2011	[11]	TP53, BRCA1, BRCA2 (frequent)<br>BRAF, PIK3CA, KRAS, NRAS, NOTCH3, FOXM1 (rare)
Ahmed et al. 2010	[16]	TP53
Roh et al. 2010	[17]	TP53, PTEN, PAX2
Yang et al. 2011	[18]	BRCA1, BRCA2
Vaughan et al. 2011	[19]	CCNE1, PIK3CA, RAS, HER2, TP53, BRCA, VEGF
Caslini et al., 2006	[20]	GATA2
Ardighieri et al. 2014	[21]	BRAF, KRAS, Ki-67
Gillet et al. 2012	[22]	MDR1
Skirnisdottir et al. 2004	[12]	P53, EGFR

Table S2. The relationship between molecular markers and clinicopathologic characteristics

Characteristics	Number (%) of subjects									
	High or absence of P53	High VEGF	High HER2	High KRAS	Low PTEN	Low BRCA1	Low BRCA2	High NOTCH3	Low BRAF	High EGFR
Parity	(P=0.042)*	(P=0.562)	(P=0.776)	(P=0.487)	(P=0.395)	(P=0.294)	(P=0.266)	(P=0.280)	(P=0.109)	(P=0.676)
0-1	59(48.0)	57(48.3)	31(54.4)	40(45.5)	35(42.2)	33(57.9)	38(55.9)	46(51.1)	56(48.7)	35(53.0)
2-3	57(46.3)	49(41.5)	22(38.6)	42(47.7)	37(44.6)	22(38.6)	21(30.9)	39(43.3)	43(37.4)	26(39.4)
>3	7(5.7)	12(10.2)	4(7.0)	6(6.8)	11(13.2)	2(3.5)	9(13.2)	5(5.6)	16(13.9)	5(7.6)
Peritoneal metastasis	(P=0.027)*	(P=0.947)	(P=0.079)	(P=0.071)	(P=0.065)	(P=0.784)	(P=0.289)	(P=0.287)	(P=0.804)	(P=0.109)
yes	79(64.2)	66(55.9)	39(68.4)	57(64.8)	55(66.3)	33(57.9)	42(61.8)	55(61.1)	65(56.5)	44(66.7)
no	44(35.8)	52(44.1)	18(31.6)	31(35.2)	28(33.7)	24(42.1)	26(38.2)	35(38.9)	50(43.5)	22(33.3)
FIGO stage	(P=0.024)*	(P=0.422)	(P=0.252)	(P=0.011)*	(P=0.221)	(P=0.231)	(P=0.352)	(P=0.199)	(P=0.089)	(P=0.625)

I	33(26.8)	41(34.7)	18(31.6)	18(20.5)	20(24.1)	15(26.3)	17(25.0)	26(28.9)	38(33.0)	18(27.3)
II	17(13.8)	13(11.0)	2(3.5)	11(12.5)	11(13.3)	12(21.1)	11(16.2)	11(12.2)	17(14.8)	9(13.6)
III	66(53.7)	59(50.0)	33(57.9)	53(60.2)	48(57.8)	26(45.6)	38(55.9)	46(51.1)	54(47.0)	37(56.1)
IV	7(5.7)	5(4.2)	4(7.0)	6(6.8)	4(4.8)	4(7.0)	2(2.9)	7(7.8)	6(5.2)	2(3.0)
Histotype	(P=0.114)	(P=0.722)	(P=0.171)	(P=0.390)	(P=0.562)	(P=0.582)	(P=0.273)	(P=0.146)	(P=0.045)*	(P=0.041)*
serous	87(70.8)	85(72.0)	36(63.2)	60(68.2)	52(62.5)	39(68.4)	46(67.6)	59(65.6)	80(69.5)	42(63.6)
mucinous	13(10.6)	11(9.3)	4(7.0)	9(10.2)	8(9.6)	6(10.5)	9(13.3)	7(7.8)	13(11.3)	4(6.1)
endometrioid	10(8.1)	9(7.6)	5(8.8)	9(10.2)	9(11.1)	5(8.8)	9(13.3)	11(12.1)	10(8.7)	9(13.6)
clear cell	4(3.3)	6(5.1)	6(10.5)	6(6.9)	7(8.4)	5(8.8)	2(2.9)	5(5.6)	4(3.5)	4(6.1)
differentiated	9(7.2)	7(6.0）	6(10.5)	4(4.5)	7(8.4)	2(3.5)	2(2.9)	8(8.9)	8(7.0)	7(10.6)
Grade	(P=0.068)	(P=0.921)	(P=0.245)	(P=0.016)*	(P=0.054)	(P=0.588)	(P=0.289)	(P=0.641)	(P=0.523)	(P=0.745)
G1	22(17.9)	25（21.2）	7(12.3)	11(12.5)	9(10.8)	9(15.8)	11(16.2)	15(16.7)	21(18.2)	11(16.7)
G2	39(31.7)	42（35.6）	20(35.1)	26(29.5)	30(36.1)	20(35.1)	21(30.9)	33(36.7)	44(38.3)	24(36.4)

G3	62(50.4)	51（43.2）	30(52.6)	51(58.0)	44(53.1)	28(49.1)	36(52.9)	42(46.6)	50(43.5)	31(46.9)
Type	(P=0.002)*	(P=0.615)	(P=0.025)*	(P=0.001)*	(P=0.005)*	(P=0.048)*	(P=0.207)	(P=0.164)	(P=0.936)	(P=0.159)
I	33(26.8)	43（36.4）	11(19.3)	18(20.5)	18(21.7)	15(26.3)	20(29.4)	26(28.9)	42(36.5)	18(27.3)
II	90(73.2)	75（63.6）	46(80.7)	70(79.5)	65(78.3)	42(73.7)	48(70.6)	64(71.1)	73(63.5)	48(72.7)
Residual disease	(P=0.102)	(P=0.533)	(P=0.002)*	(P=0.066)	(P=0.112)	(P=0.777)	(P=1.000)	(P=0.336)	(P=0.631)	(P=0.168)
≤0.5 cm	86(69.9)	88(74.6）	26(45.6)	50(56.8)	44(33.7)	32(56.1)	50(73.5)	63(70.0)	83(72.2)	44(66.7)
>0.5 cm	37(30.1)	30(25.4)	31(54.4)	38(43.2)	39(66.3)	25(43.9)	18(26.5)	27(30.0)	32(27.8)	22(33.3)
Platinum resistance	(P=0.161)	(P=0.721)	(P=0.013)*	(P=0.004)*	(P=0.043)*	(P=0.190)	(P=0.281)	(P=0.280)	(P=0.853)	(P=0.180)
yes	33(26.8)	26(22.0)	26(45.6)	32(36.4)	36(43.4)	30(52.6)	23(33.8)	25(27.8)	32(27.8)	20(30.3)
no	90(73.2)	92(78.0)	31(54.4)	56(63.6)	47(56.6)	27(47.4)	45(66.2)	65(72.2)	83(72.2)	46(69.7)

*statistical significance

Supplementary Methods

The staining evaluation criteria for 15 molecular markers

P53: Nuclear staining was considered to be a positive P53 reaction. The TP53 mutation was associated with a strong staining and a diffuse (in greater than 60% fraction of cells) pattern (++ - +++) or the complete absence (-) of P53 expression. The presence of rare and normal positive nuclei were identified as wild type TP53 [24].

KRAS, FOXM1, GATA2, VEGF, Ki67, EGFR, MDR1, CCNE1, NOTCH3, PTEN, BRAF: Staining results were scored on the positive fraction of stained cells (0: 0-9% tumor cells stained; 1: 10-24% tumor cells stained; 2: 25-50% tumor cells stained; 3: >50% tumor cells stained) and the intensity of staining (0: none; 1: light; 2: moderate; 3: strong). The fraction and intensity scores were multiplied to obtain the final score. Molecular expression was measured using the final score: >6(+++), 5-6(++), 3-4(+), 1-2(±), 0(-). KRAS, FOXM1, GATA2, Ki67 and CCNE1 were all predominantly found in the nucleus. VEGF and EGFR were identified as positive with the appearance of brown particles in the plasma membrane. MDR1, BRAF and NOTCH3 were expressed in the cytoplasm [12,25-28]. The KRAS mutation had strong staining (score: 5-9) in the tumor cell nuclei, while wild-type KRAS showed weak staining. PTEN expression was shown in cell nuclear and cytoplasm. PTEN was considered to be mutation with score of 0-6 and wild-type with score >6 [29]. Negative (score: 0) and weak (score: 1-2) staining was considered to be mutation BRAF, and wild-type BRAF showed moderate to strong staining (score: 3-9) [30].

HER2: 1-9% of tumor cells with membrane staining was considered to be HER2 ±, 10-30% staining considered to be HER2 1+ and more than 30% staining considered to be HER 2+ [31].

BRCA was expressed in the cell nuclear. If there was a complete absence of staining compared with the presence of a positive internal control, weak staining in 5-10% of the tumor cell nuclei in the presence of a moderate to strong internal positive control, or staining in less than 5% of the tumor cells, a BRCA mutation was declared. BRCA was considered to be normal when staining occurred in more than 10% of the tumor cell nuclei or moderate staining in 5-10% of the tumor nuclei with a moderate internal control [32].

Figure S1. Single immunostaining of 15 molecular markers in epithelial ovarian cancer. (A) BRAF expression, (B) CCNE1 expression, (C) BRCA1 expression, (D) BRCA2 expression, (E) EGFR expression, (F) FOXM1 expression, (G) GATA2 expression, (H) HER2 expression, (I) Ki67 expression, (J) KRAS expression, (K) MDR1 expression, (L) NOTCH3 expression, (M) P53 expression, (N) PTEN expression, (O) VEGF expression. (original magnifications × 200, except for HER2 × 400)

Figure S2. Kaplan-Meier overall survival analyses of 15 molecular markers in epithelial ovarian cancer. The overall survival was compared between different patient groups (low expression vs. high expression). 10 molecular markers were significantly associated with overall survival.
